# Supplementary material for: Emulsion Surimi Gel with Tunable Gel Properties and Improved Thermal Stability by Modulating Oil Types and Emulsification Degree
Source: Foods. 2022 Jan 11;11(2):179. doi: 10.3390/foods11020179 (PMC8774618; doi:10.3390/foods11020179)
Supplement: Supplementary file 1 [file foods-11-00179-s001.zip › foods-1486628-supplementary.pdf]

2.Confocal laser scanning microscopy (CLSM)

The distribution of proteins in samples was assessed by confocal laser scanning microscope (LSM710, Zeiss, Germany), in the fluorescence mode. Aliquots (40 ml) of staining solution containing 0.01 % (w/v) Rhodamine B (protein dye) was added to 1 g of sample with gentle stirring, and afterward, the samples were washed by distilled water to remove excessive stain. Stained samples were immediately mounted onto a single-well slide, and a coverslip was placed carefully, to avoid any air bubbles between the sample and the coverslip. Samples were observed under the mi croscope, using 40 × magnification, and the excitation was per formed using laser beam at 559 nm. The emission of Rhodamine B was measured at between531 and 703 nm. Twenty images of each sample were obtained, and all experiments were performed in triplicate.

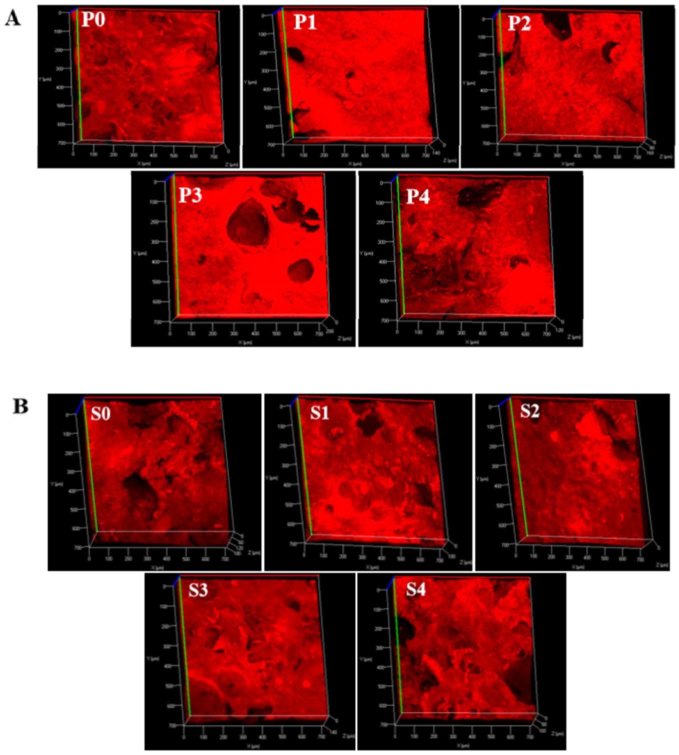

Figure S1. The microstructures of surimi gel emulsified by perilla seed (A) oil and soybean oil (B) subjected to heating treatments (40 ×).
